# Time for a globally unified chronic HBV terminology?

**DOI:** 10.1016/j.jhepr.2025.101570

**Published:** 2025-08-29

**Authors:** Su Wang, Catherine Freeland, Seng Gee Lim, Hailemichael Desalegn, Chari Cohen, Harry L.A. Janssen

**Affiliations:** 1 Cooperman Barnabas Medical Center, Rutgers New Jersey Medical School, NJ, United States; 2 Hepatitis B Foundation, PA, United States; 3 National University Hospital of Singapore, Singapore; 4 St. Paul’s Hospital, Addis Ababa, Ethiopia; 5 Toronto Center for Liver Disease, Canada; 6 Erasmus MC Rotterdam, Department of Gastroenterology and Hepatology, the Netherlands

**Keywords:** Hepatitis B Terminology, Hepatitis B Care, Liver Cancer, Hepatitis B, Viral Hepatitis, Hepatitis Guidelines, Hepatitis B Phase, Hepatitis B Drug Development, Hepatitis B Cure, Hepatitis B Natural History

## Abstract

The terminology used to describe chronic hepatitis B (CHB) infection remains inconsistent and fragmented across liver societies, clinical settings, and research domains. This lack of alignment poses barriers to care, complicates clinical trial design, and can generate confusion among providers, people living with hepatitis B, and researchers. This article examines the impact of discordant CHB infection terminology on care delivery and research, highlighting specific challenges with commonly used terms, such as “immune tolerant,” “indeterminate” or “grey zone”, as well as with terms used for hepatitis B surface antigen loss, including “resolved infection”, “occult infection” or “functional cure.” Although recent guidelines have moved towards simplification, global uniformity remains lacking, particularly regarding definitions of disease phases and thresholds for initiating treatment. We call for alignment of terminology to improve care, increase treatment uptake, enhance patient engagement, and accelerate HBV research and elimination efforts. We propose a multistakeholder consensus process to create a unified and practical nomenclature that distinguishes between terminology for clinical care and terminology for research and drug development. We also call for intentional inclusion of people with lived experience in this process to ensure the language used is meaningful, empowering, and stigma-free. With the HBV field on the cusp of transformative therapies and simplified treatment algorithms, now is the time to harmonise the language we use. A globally unified chronic HBV infection terminology stands to enhance access to care, improve comparability of research data, and strengthen collaboration across the HBV community – all of which are critical to accelerating progress towards hepatitis B elimination.

## Graphical abstract

**Figure:**
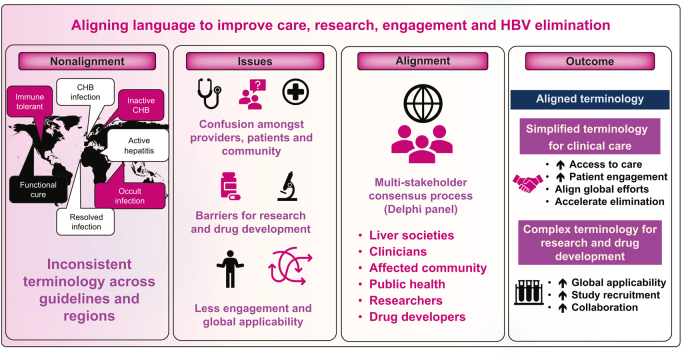

**Key points**

• Current terminology used to describe chronic hepatitis B infection phases is variable and inconsistent amongst liver societies and other experts.
• Non-aligned terminology has a negative impact on care, research, and people living with chronic HBV infection.
• Specifically, terms like “immune tolerant” and “indeterminate” or ‘grey zone” pose challenges and lead to confusion among providers, people living with hepatitis B and the wider community.
• Terms for HBsAg loss are also varied and include “resolved HBV”, “occult infection” and “functional cure”.
• There is a pressing need for a consensus effort to align terminology to work towards unified elimination efforts, as our understanding of chronic HBV evolves.
• Consensus development should be a multistakeholder effort involving clinicians, researchers, drug developers, public health specialists and the affected community.

## Background: HBV terminology variability and general impact

The course of chronic hepatitis B (CHB) infection is influenced by complex virological, host, and environmental factors.^1^ Expert groups have created terminology to describe disease phases using viral proteins (hepatitis B surface antigen [HBsAg] and hepatitis B e antigen [HBeAg]), viral load (HBV DNA), and alanine aminotransferase (ALT).^2^ However, these phase classifications vary across liver societies, using different terms and cut-offs, making them difficult to apply in clinical practice – especially in resource-limited areas. Determining disease phase often requires extensive, repeated testing, which can make management and treatment more difficult for providers.^3^ In many low- and middle-income countries, essential diagnostics such as HBeAg and HBV DNA, which are needed for classification, are not readily available, leading to treatment delays and acting as a major barrier to timely care. The lack of unified terminology also hinders coordination across clinical care, research, and public health efforts, affecting stakeholders from clinicians to people living with hepatitis B (PLWHB).

Practical approaches are needed to support global HBV elimination and prevent liver cancer. Efforts to decentralise CHB infection care so that non-specialists can care for the over 250 million estimated to be living with CHB infection will require simplification of terminology and treatment approaches. Harmonising terminology is essential to strengthen collaboration between scientific, clinical, and lived experience communities, helping bridge gaps and streamline efforts to move the field forward.

This review examines 1) the impact of non-aligned terminology on care, research and the affected community; 2) areas of CHB infection terminology that are problematic; 3) our changing understanding of the virus and its impact on terminology; and 4) the pressing need for a consensus effort to harmonise terminology, along with considerations for next steps.

## Summary of current terminology

The natural history of CHB infection has been described in phases, though an individual may not go through all phases within their lifetime and the duration in each phase varies. Phase terminology has sought to capture distinct characteristics and patterns of HBV in its progression.^1^ CHB infection can progress both sequentially and non-sequentially, and people may move back and forth between phases. Close monitoring is necessary to detect phase transitions. These phases are characterised by their immunological features, virology, biochemistry and histology. Liver societies have historically defined 3-5 distinct phases, though the term “indeterminate phase” has become a broad catch-all when criteria for the defined phases are not met. Despite recent guideline updates in 2024 (WHO^4^) and 2025 (EASL^5^ and AASLD, pending publication), terminology has not been significantly simplified or harmonised across societies. Discrepancies and challenges in terminology can be seen in Table 1,[4], [5], [6], [7] which provides a brief overview of the terminology used by three large international liver societies – American Association for the Study of Liver Disease (AASLD), European Association for the Study of the Liver (EASL), Asian Pacific Association for the Study of the Liver (APASL) – and the World Health Organization (WHO). Other regional or country-based society guidelines have their own phase nomenclature, which should be considered in efforts to align HBV terminology globally.[8], [9], [10], [11]

**Table 1:** HBV terminology and characteristics by liver societies & WHO.

Phase∗	AASLD 2016	EASL 2025	APASL 2015	WHO 2024	Key features
1	Immune tolerant	HBeAg-positive chronic HBV infection	Immune tolerant	HBeAg-positive infection	HBeAg+, high HBV DNA (>10^7^ IU/ml), normal ALT, minimal/no liver damage
2	HBeAg-positive immune-active phase (2018 guidance: immune-active chronic HBV infection)	HBeAg-positive chronic hepatitis B	Immune reactive (immune-active/immune clearance/HBeAg-positive CHB/HBeAg clearance phase)	HBeAg-positive disease	HBeAg+, high HBV DNA, elevated ALT, active liver inflammation
3	Inactive chronic HBV infection phase	HBeAg-negative chronic HBV infection	Low replicative chronic HBV infection	HBeAg-negative infection	HBeAg-, low HBV DNA (<2,000 IU/ml), normal ALT, low liver activity
4	HBeAg-negative immune reactivation phase (2018 guidance: immune-active chronic HBV infection)	HBeAg-negative chronic HBV infection	Reactivation phase or HBeAg-negative/anti-HBe positive chronic HBV infection	HBeAg-negative disease	HBeAg-, persistent or fluctuating moderate/high HBV DNA, elevated ALT, liver inflammation and damage possible
5	Resolved infection	HBsAg loss (HBsAg -, anti-HBc+, HBsAb +/-)	Resolved infection	Occult hepatitis B	HBsAg-, very low/undetectable HBV DNA, signifies viral clearance or immune control
HBV profiles that fall outside defined phases	Grey zone	Avoid using indeterminate phase term; additional categories for research purposes	Indeterminate phase	Grey zone or Indeterminate	Does not fit criteria of defined phases; variable serology/ALT/HBV DNA

AASLD, American Association for the Study of Liver Diseases; ALT, alanine aminotransferase; APASL, Asian Pacific Association for the Study of the Liver; CHB, chronic hepatitis B; EASL, European Association for the Study of the Liver; HBeAg, hepatitis B e antigen; HBsAb, hepatitis B surface antibody; HBsAg, hepatitis B surface antigen; WHO, World Health Organization.

∗ Numbering used here for organizational purpose, only EASL explicitly labels the phases by number (1-4 per the updated 2025 guidelines).

## Impact of non-aligned terminology on research and drug development

Non-aligned terminology and definitions present significant challenges for research, especially in global contexts. The varying criteria can create barriers to clinical trial enrollment. Liver societies also have different cut-offs for treatment initiation in their guidelines, meaning that disease phase may differ amongst patients who are started on therapy and enrolled in studies. Thus, research initiatives may be difficult to adapt across sites or scale globally due to differing definitions and treatment criteria.

Challenges in replicating and implementing clinical research programmes can limit uptake and hinder programme expansion. Given the limited resources available within the HBV space and significant costs of drug development, particularly when enrolling multiple subgroups, collaboration and harmonisation are essential to improve efficiencies and global applicability. Due to the risk of fragmented investment caused by complexities in the field, a coordinated effort is essential to align terminology, concentrate resources, and amplify impact.

## Impact of non-aligned terminology on clinical care and delivery

Current treatment eligibility depends on identifying when patients are in an immunoactive stage, which requires numerous tests (HBeAg, HBV DNA, ALT) over time. These tests are not accessible in many low- and middle-income countries^11^ and thus, this approach poses a significant barrier to treatment. Decentralisation of care is essential to reach global elimination targets for HBV; however, many healthcare professionals, especially general practitioners, have difficulty interpreting the various biomarkers involved in HBV.^12^^,^^13^ Liver societies have varying HBV DNA and ALT criteria, so the determination of eligibility depends on which guidelines one follows. Interpretation of HBV screening tests is already difficult, and the variability of cut-offs and normal ALT thresholds adds to the challenge of decentralised care. The need for longitudinal monitoring to determine when a patient enters the “active” phase has also led to delayed treatment and can contribute to loss to follow-up.^14^

Despite progress in HBV elimination efforts, concerning gaps remain in diagnosis and treatment hindering the achievement of WHO’s 2030 elimination targets.^15^ Only 13% of PLWHB have been diagnosed and only 2.6% have been treated.^15^ Many PLWHB who are treatment eligible, by AASLD and EASL guidelines, are not on therapy and studies have shown that the more tests needed for assessment, the fewer people are treated.^16^^,^^17^

Decentralising care from specialists to primary care will be necessary to achieve HBV elimination; however, general practitioners report that the complexity of CHB infection is a barrier to delivering care.^12^ Studies often highlight low practitioner knowledge on management as well as confusion on the correct approaches for HBV care.[18], [19], [20] Inadequate understanding of CHB infection is associated with decreased adherence to care and treatment protocols, increased discriminatory behaviours toward affected individuals, and the perpetuation of stigma, collectively contributing to adverse health outcomes.[21], [22], [23], [24], [25] Practitioners also cite the lack of simplified guidelines as a barrier to providing care.^26^^,^^27^

Simplifying CHB infection management is necessary to achieve elimination, and aligning terminology is key to this process and ensuring global harmonization. Recent guidelines with simplified approaches, such as the WHO’s 2024 HBV treatment guidelines,^10^ have removed one test, HBeAg, from their previous treatment algorithms (except in pregnancy where it can be used in lieu of HBV DNA when not accessible). WHO guidelines use four criteria to determine treatment eligibility, which include evidence of fibrosis (APRI [aspartate aminotransferase-to-platelet ratio index] >0.05), an HBV DNA >2,000 IU/ml, presence of coinfections (or comorbidities or other risk factors) or persistently abnormal ALT levels.^4^ Other treatment algorithms have taken similar steps to simplify treatment approaches and enable decentralised care (Guidance for the Primary Care Provider, the Chinese guidelines and the Simplified Approach Hepatitis B Algorithm).^8^^,^^26^^,^^28^

Another set of terminology that should be aligned is the ICD codes for HBV infection. The WHO publishes ICD codes, a system used to code and classify medical diagnoses, which are used in billing and attached to visits, laboratory tests, procedures, insurance reimbursements and death certificates. ICD-10 codes are often used to track healthcare utilisation and are even applied to estimate global hepatitis disease burden and related mortality.^29^ The current CHB infection categories in ICD-10 are B18.0 (Chronic viral hepatitis B with delta agent), B18.1 (Chronic viral hepatitis B without delta agent), B19.1 (unspecified viral hepatitis B without coma) and B19.11 (unspecified viral hepatitis with hepatic coma). The subcategories of chronic HBV infection, *e.g.* with or without “delta” and “coma”, affect a minority of patients with HBV and could be replaced with more clinically relevant subcategories, such as abnormal liver enzymes, fibrosis or cirrhosis.

## Impact on people living with hepatitis B

Nomenclature impacts people with lived experience. Unclear CHB infection terminology can confuse patients and negatively impact care. Many are told by physicians that they are “HBV carriers” or that their CHB infection is “not active” and that they “do not need medication, so they do not need to worry”, which often leads to a belief that follow-up or treatment is not necessary.^30^ The important messages of needing to continue surveillance and that CHB infection is a dynamic disease may not be conveyed by all providers or may not be understood by patients. Patients may perceive that once they are told they do not need medication, CHB infection is not something they need to worry about.^30^ In the scenario of HBsAg loss, the term “resolved infection” may imply complete eradication to people, and they may be unaware of the persistence of covalently closed circular DNA (cccDNA) and the ongoing risk of HBV reactivation.

Terms like “immune tolerant”, “indeterminate”, “HBV infection” *vs.* “hepatitis” or “immune active” are often not explained, leaving patients focused only on whether they need medication and future monitoring or follow-up.^31^^,^^32^ Patients also may not understand why they are not offered treatment with very high HBV DNA, as in the first phase, but then it is indicated when their HBV DNA level is lower. Research has found that many who remain outside of treatment guidelines have higher rates of liver cancer than those with compensated cirrhosis and HBV DNA suppression achieved through therapy.^33^^,^^34^ This research calls into question the current guidelines and their approaches to treatment, which can leave people in the indeterminant or grey zone phases at risk. It is worth considering the message we send that intervention is indicated only when there is evidence of inflammation, rather than prioritising prevention of inflammation and damage.

Barriers to care and challenges to accessing specialists and diagnostics should be considered. Many PLWHB come from vulnerable communities and face challenges, including language barriers, lack of insurance, management and treatment costs, access to diagnostics, stigma, discrimination, and other psychosocial and mental health challenges.^32^^,^^33^^,^^35^^,^^36^ Long-term laboratory-based monitoring can be a challenge, and studies have found lower rates of adherence to guideline-based monitoring amongst patients who are not on medication.^37^

## Problematic term: individuals falling outside phase definitions (indeterminate or grey zone)

When criteria are not met for other categories, patients are often labelled as being in the “indeterminate” or “grey zone”. This is problematic because this is a heterogenous group, including both HBeAg-positive and HBeAg-negative patients with a range of viral loads.^38^ They may not portend the same clinical risk or outcome, yet are all labeled as indeterminate because they fall outside of current criteria for other phases and treatment thresholds. One analysis identified 10 distinct indeterminate states with unique features.^39^ Studies have shown that 30-50% of people with CHB infection may be in an indeterminate phase,^38^^,^^40^^,^^41^ and often remain there, with one study showing that 53% of patients remained indeterminate over 10 years of follow-up.^41^ Thus, the indeterminate states may warrant their own phenotypes rather than be considered in-between stages of our current categories, as growing evidence shows these are natural long term states of the virus. The use of the “grey” and “indeterminate zone” labels also has unintended consequences for both providers and patients. Providers may be less proactive about treating these patients thinking they will transition, and patients may believe they are at low risk because they are not being treated, yet studies show that many people with liver cancer fall into these indeterminate states.^41^ The 2025 EASL guidelines do address the problematic language of indeterminate phase or grey zone, and they propose more refined terminology for research purposes.^5^

## Controversial term: immune-tolerant phase

The first phase, characterised by high HBV DNA and minimal inflammation, is a controversial area. This has been referred to as the immune-tolerant (IT) phase, although the term has fallen out of favour with many groups. The HBV DNA criteria for IT ranges from >20,000 according to APASL to >1,000,000 IU/ml according to AASLD, with normal liver enzyme levels (Table 2). In the EASL 2012 guidelines, it was described as high viral load, without an HBV DNA threshold. The term was renamed by EASL in 2017 and kept in the 2025 guidelines as “*Phase 1:* HBeAg-positive chronic HBV infection”.^5^^,^^42^ Many studies show that individuals classified as IT by laboratory tests (high HBV DNA, normal ALT) may have evidence of histologic activity or fibrosis on liver biopsy.^43^^,^^44^ Additionally, because it is not devoid of immunologic activity – such as clonal expansion, B-cell responses, and T-cell depletion^45^ – some have termed it a “high replication, low inflammation stage” or “non-inflammatory HBeAg-positive” phase.^46^ Others argue that immune tolerance to the virus occurs in multiple stages of CHB infection, contributes to viral chronicity, and therefore should not be attributed to a single phase.^47^

**Table 2:** HBV first phase nomenclature definition and management.

Clinical Item	AASLD 2016	APASL 2015	EASL 2025	WHO 2024
Name	Immune tolerant	Immune tolerant	HBeAg-positive chronic HBV infection	HBeAg-positive infection (immune tolerant)
Definition	HBV DNA >1 million IU/ml ALT normal	Persistence of HBeAg-positive infection without significant ongoing neuroinflammatory disease HBV DNA >20,000 IU/ml ALT 1-2x the upper limit of normal	Persistently normal ALT and high HBV DNA levels	>10^7^ with normal or near- normal ALT, minimal neuroinflammatory change on liver biopsy, no or slow progression to fibrosis and low spontaneous HBeAg loss.
Monitoring	Every 6 months	Every 3 months	3-6 months (or until treatment is initiated, after initial phase, the monitoring frequency can be adjusted to 6-12 months depending on disease phase)	At least annually for those on and off treatment
Treatment	In patients >40 years w/normal ALT and elevated HBV DNA >1,000,000 IU/ml and liver biopsy showing significant necroinflammation or fibrosis (F2)	If biopsy shows moderate/severe inflammation (A3) or significant fibrosis (F2)	May be treated if they are older than 30 years or family history of HCC/cirrhosis	If significant fibrosis >F2 or cirrhosis (using non-invasive tests such as APRI) *OR* HBV DNA >2,000 IU/ml with abnormal ALT *OR* Presence of any coinfection, family history, immune suppression, comorbidities or extrahepatic manifestations *OR* Persistently abnormal ALT alone
ALT cut-off	30 IU/ml men 25 IU/ml women	40 U/L	ALT>ULN	30 U/L for men and boys 19 U/L for women and girls

AASLD, American Association for the Study of Liver Diseases; ALT, alanine aminotransferase; APASL, Asian Pacific Association for the Study of the Liver; APRI, aspartate aminotransferase-to-platelet ratio index; EASL, European Association for the Study of the Liver; HBeAg, hepatitis B e antigen.

## Call for alignment: consideration and next steps

There is a pressing need to unify our CHB infection nomenclature globally to improve research and care efforts around the world and provide more clarity to relevant stakeholders, including the patient community. One should consider the benefits of consensus over the current siloed approach, in which liver societies and professional bodies each develop their own nomenclature. Input is needed from multiple stakeholders, including specialists (hepatologists, infectious disease providers) and non-specialist clinicians, researchers, including virologists and immunologists, regulatory agencies, and the public health and patient communities, to develop global consensus. Consensus could assist with global HBV elimination by decreasing confusion about clinical management and enabling simplification to increase access, while maintaining complexity for research and drug development.

### Consensus development

The Delphi technique has been widely used to build consensus on clinical issues and to develop, describe and evaluate clinical guidelines or tools.^48^ The Delphi panel is asked for their opinion on a relevant issue. Their collective responses are summarised and presented, and this process is repeated over several rounds. It has been used in many disease states,^49^ and in 2023, liver societies and stakeholders, including people with lived experience, came together to update the nomenclature for “non-alcoholic fatty liver disease or NAFLD”. This resulted in the new term “metabolic dysfunction-associated steatotic liver disease or MASLD”, which is expected to create new opportunities for natural history and drug development research, as well as improved strategies for diagnosis, monitoring, care and treatment. The Delphi process, as used to develop the EASL HBV guidelines, could be an effective strategy to align HBV global experts and stakeholders to harmonise language and thereby improve care and research.

### Affected community involvement

Of primacy, CHB infection nomenclature must be meaningful and helpful to the affected community. Terminology is important for clinicians and researchers but should also be useful and understandable to PLWHB, so they can actively participate in their own care.^50^ Language should be inclusive and non-stigmatising so that it does not present a barrier for people to access and stay in care.^51^^,^^52^ Engaging empowered communities and civil society is one of the five strategic directions of WHO’s Global Health Sector Strategy to effectively achieve hepatitis elimination.^51^ As an example, the WHO’s HBV guideline development group included people with lived experience and representatives from the community, affirming the important principle “nothing about me without me” and the need for intentional engagement.^10^^,^^53^^,^^54^ Harmonisation of CHB infection terminology should also incorporate the perspectives of the affected community and actively involve PLWHB.

### Nomenclature considering newer diagnostics and evolving understanding of HBV

Newer diagnostics for HBV, such as quantitative HBsAg (qHBsAg), HBV RNA and core-related Ag (HBcrAg), could improve our understanding of the disease, though their utility is still being explored.^55^ Of these, qHBsAg is commercially available in some areas and reflects overall transcriptional activity of the virus, including that from both cccDNA and integrated DNA within liver cells. These additional tests may contribute to our knowledge of the natural history of HBV and the resulting terminology. For patients on nucleoside analogues who have undetectable viral load, qHBsAg provides additional information on viral activity in the liver. HBV RNA and HBcrAg reflect transcriptionally active cccDNA more specifically than qHBsAg, which is important for the development of new compounds aimed at cure of HBV. In the 2025 EASL guidelines, a proposal to add HBcrAg and HBV RNA to standard test profiles results in 11 different classifications of CHB infection, and such nuances can be useful for research and drug discovery.^5^

### Separating terminology for care from terminology for research and drug development

It is important that nomenclature for frontline clinical settings be simpler to improve access and retention to care, while the nomenclature for research and more specialised settings can remain more complex where nuances of the phases are needed.

Nomenclature for research and clinical trial development may require more complex testing, so as not to lose the ability to more precisely evaluate phases as needed for treatment initiation, close monitoring and to determine if endpoints are met. In contrast, simplified nomenclature and guidelines for care and treatment will enable a frontline provider to take on hepatitis B care.^18^^,^^56^^,^^57^

### HBsAg loss and endpoint terminology

As the research for HBV cure moves forward, aligning clinical terminology with drug development endpoints is essential. Currently, the terminology used for HBsAg loss is non-aligned and includes “resolved infection,” “occult infection,” “seroclearance,” and in drug development, “functional cure” (Table 3).[5], [7], [8]^,^^58^^,^^59^ Those who clear HBV naturally have often been referred to as “occult” or “resolved”, while those who achieve cure on medication have been called “functionally cured” after 24 weeks off therapy. People with acute HBV infection who recover are also considered to have “resolved infection”.^60^

**Table 3:** Nomenclature and definitions for loss of HBsAg state(s).

	AASLD 2016	APASL 2015	EASL 2025	WHO 2024	2022 AASLD-EASL HBV-HDV Treatment Endpoint Conference
Term	Resolved infection	Occult infection	HBsAg loss	Occult HBV infection	Functional cure/HBsAg seroclearance
Defini-tion	Sustained HBsAg loss with undetectable HBV DNA (AASLD 2018- clearance of HBsAg with acquisition of HBsAb)	HBsAg -, anti-HBc+, HBsAb +/-	Previous HBV infection with current state of HBsAg-, with or without anti-HBs+	HBsAg is not detectable in the blood HBV DNA- Low at detection limit	Sustained HBsAg loss and HBV DNA less than the LLOQ 24 weeks off treatment.

AASLD, American Association for the Study of Liver Diseases; ALT, alanine aminotransferase; APASL, Asian Pacific Association for the Study of the Liver; EASL, European Association for the Study of the Liver; HBc, hepatitis B core; HBsAb, hepatitis B surface antibody; HBsAg, hepatitis B surface antigen; LLOQ, lower limit of quantitation.

Terms such as “functional cure” and “sterilising cure” need to be explored for their impact on individuals with lived experience.^61^^,^^62^ Studies in the HIV community looking at the same terms show that ”functional cure“ is not easily understood and evokes negative connotations among people with HIV, and “sterilising” is associated with disinfection or cleansing and is associated with infertility.^61^^,^^62^ PLWHB may differ, an initial assessment from a 2020 global survey showed that among 1,707 respondents, 61% of respondents felt that “functional cure” was the most appropriate and meaningful term.

There can be confusion about whether HBsAg loss through treatment is described using the same terminology as when naturally occurring. “Functional cure” has specifically been defined as HBsAg loss 24 weeks off therapy.^58^ However, for spontaneous HBsAg loss, “resolved” or “occult” infection have been more traditionally used. Without clear integration of these terms, many who naturally clear HBsAg are also being labelled “functionally cured.” This can lead to confusion for patients and providers and those in drug development.^63^

One proposal is a “sustained control” term (instead of inactive carrier phase or partial cure), “resolved chronic infection” (instead of functional cure), and “cure” (for sterilising cure).^64^ “Sustained virologic response” could also be an important milestone for finite therapy in drug development and has a precedence in HCV curative treatments. However, there is a counter argument for maintaining the “functional cure” terminology. “Cure” is a powerful term that gives hope to PLWHB and is much anticipated in the community. For drug development, it is an easily understood term and has energised the field. As we discuss potential changes in therapeutic endpoint terminology, it is essential to include PLWHB as partners in these discussions.

Simplistic and consistent language is needed to give providers, drug developers and people with lived experience clarity. Future finite and curative therapies could also facilitate simplified treatment strategies, as occurred with hepatitis C, making determination of phases less important.^59^

## Conclusion

**Fig.1:**
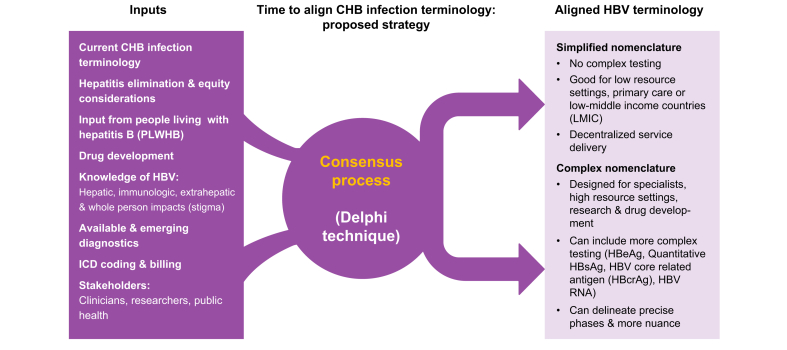
Strategy to align CHB infection terminology. CHB, chronic hepatitis B.

As the landscape and understanding of hepatitis B evolve, so must the language and terminology used to describe it. Current CHB infection nomenclature is fragmented, inconsistent across global guidelines, and often too complex for clinical application, leading to confusion among providers and patients and posing challenges in research and drug development. Simplifying and aligning terminology, while distinguishing between clinical and research needs is essential to improving diagnosis, treatment, and monitoring. Involving people with lived experience and adopting inclusive, accurate, and empowering language will not only reduce stigma but also improve engagement in care and support global HBV elimination goals. A coordinated effort is urgently needed to create a unified, practical and person-centred language for the hepatitis B field. We call on liver societies and stakeholders to come together and develop a consensus to align terminology to improve care and accelerate the development of new therapies for the 300 million people living with chronic hepatitis B infection (Fig. 1).

## Abbreviations

AASLD, American Association for the Study of Liver Diseases; ALT, alanine aminotransferase; APASL, Asian Pacific Association for the Study of the Liver; cccDNA, covalently closed circular DNA; CHB, chronic hepatitis B; EASL, European Association for the Study of the Liver; HBcrAg, hepatitis B core-related antigen; HBeAg, hepatitis B e antigen; HBsAg, hepatitis B surface antigen; IT, immune tolerant; PLWHB, people living with hepatitis B; qHBsAg, quantitative hepatitis B surface antigen; WHO, World Health Organization.

## Financial support

No financial support was involved in the development of this manuscript.

## Authors’ contributions

CF: Initial Draft, Conceptualization, Content Expert, Review, Editing, Finalization of draft. SW: Initial Draft, Conceptualization, Content Expert, Review, Editing, Finalization of draft. SGL: Content Review, Editing, Content Expert, Final Draft. HD: Content Review, Content Expert, Editing, Final Draft. CC: Content Review, Content Expert, Editing, Final Draft. HJ: Content Review, Content Expert, Editing, Final Draft.

## Conflict of interest

SW reports grant support from Gilead Sciences, paid to institution, CC and CF reports grants from Gilead sciences, GSK, Vir biotechnology, Dynavax, Roche paid to institution. SL is on advisory boards for GlaxoSmithKline, Roche, Arbutus, Assembly, AusperBio, Gilead Sciences, Sysmex, Grifols, Aligos and Abbott and speakers bureaus for GlaxoSmithKline, Gilead Sciences, Abbott, Sysmex, and Roche. He receives educational/research funding from Abbott, Roche, Sysmex, Gilead Sciences. HD declares no conflicts. HJ reports grants from AbbVie, Aligos, Arbutus, Gilead Sciences, GSK, Janssen, Roche, Vir-Bio.

Please refer to the accompanying ICMJE disclosure forms for further details.

## References

[1] Croagh C.M.N., Lubel J.S. (2014). Natural history of chronic hepatitis B: phases in a complex relationship. World J Gastroenterol.

[2] Yim H.J., Kim J.H., Park J.Y. (2020). Comparison of clinical practice guidelines for the management of chronic hepatitis B: when to start, when to change, and when to stop. Clin Mol Hepatol.

[3] McMahon B.J. (2009). The natural history of chronic hepatitis B virus infection. Hepatology.

[4] (2024). Guidelines for the prevention, diagnosis, care and treatment for people with chronic hepatitis B infection.

[5] European Association for the Study of the Liver (2025 May 8). EASL Clinical Practice Guidelines on the management of hepatitis B virus infection. J Hepatol.

[6] Terrault N.A., Bzowej N.H., Chang K.M. (2016). AASLD guidelines for treatment of chronic hepatitis B. Hepatology.

[7] Sarin S.K., Kumar M., Lau G.K. (2016 Jan). Asian-Pacific clinical practice guidelines on the management of hepatitis B: a 2015 update. Hepatol Int.

[8] You H., Wang F., Li T. (2023). Guidelines for the prevention and treatment of chronic hepatitis B (version 2022). J Clin Transl Hepatol.

[9] Coffin C.S., Fung S.K., Ma M.M. (2012).

[10] Chien R.N., Kao J.H., Peng C.Y. (2019 Jan). Taiwan consensus statement on the management of chronic hepatitis B. J Formos Med Assoc.

[11] Jaquet A., Muula G., Ekouevi D.K. (2021). Elimination of viral hepatitis in low and middle-income countries: epidemiological research gaps. Curr Epidemiol Rep.

[12] Howell J., Seaman C., Wallace J. (2023). Pathway to global elimination of hepatitis B: HBV cure is just the first step. Hepatology.

[13] Kim T Van, Pham T.N.D., Phan P. (2024). Effectiveness and implementation of decentralized, community- and primary care-based strategies in promoting hepatitis B testing uptake: a systematic review and meta-analysis. EClinicalMedicine.

[14] Nguyen M.H., Wong G., Gane E. (2020). Hepat B Virus Adv Prev Diagn Ther.

[15] (2024). Global Hepatitis Report 2024 Action for Access in Low- and Middle-Income Countries.

[16] Spearman C.W., Andersson M.I., Bright B. (2013). Hepatitis B in Africa Collaborative Network (HEPSANET). A new approach to prevent, diagnose, and treat hepatitis B in Africa. BMC Glob Public Health.

[17] Ye Q., Kam L.Y., Yeo Y.H. (2022). Substantial gaps in evaluation and treatment of patients with hepatitis B in the US. J Hepatol.

[18] Xiao Y., van Gemert C., Howell J. (2022). A survey of knowledge, attitudes, barriers and support needs in providing hepatitis B care among GPs practising in Australia. BMC Prim Care.

[19] Pham T.T.H., Le T.X., Nguyen D.T. (2019). Knowledge, attitudes and medical practice regarding hepatitis B prevention and management among healthcare workers in Northern Vietnam. PLoS One.

[20] Chao S.D., Wang B.M., Chang E.T. (2015). Medical training fails to prepare providers to care for patients with chronic hepatitis B infection. World J Gastroenterol.

[21] Gambhir R., Kapoor V., Jindal G. (2013). Attitudes and awareness regarding Hepatitis B and Hepatitis C amongst health-care workers of a tertiary Hospital in India. Ann Med Health Sci Res.

[22] Freeland C., Qureshi A., Wallace J. (2024). Hepatitis B discrimination: global responses requiring global data. BMC Public Health.

[23] Thomson M.J., Lok A.S., Tapper E.B. (2018). Optimizing medication management for patients with cirrhosis: evidence-based strategies and their outcomes. Liver Int.

[24] Krousel-Wood M., Hyre A., Muntner P. (2005). Methods to improve medication adherence in patients with hypertension: current status and future directions. Curr Opin Cardiol.

[25] Aremu T.O., Oluwole O.E., Adeyinka K.O. (2022). Medication adherence and compliance: recipe for improving patient outcomes. Pharmacy.

[26] Dieterich D., Graham C., Wang S. (2023). It is time for a simplified approach to hepatitis B elimination. Gastro Hep Adv.

[27] Moussa D., Wallace J., Manski-Nankervis J.A. (2023). Assessment of a primary care e-support package of automated case finding, simplified treatment algorithm and decision support to increase hepatitis B treatment uptake in primary care clinics in Australia (SIMPLY-B Study): protocol for a pilot evaluation. BMJ Open.

[28] Tang A.S., Thornton K. (2020).

[29] Sheena B.S., Hiebert L., Han H. (2022). Global, regional, and national burden of hepatitis B, 1990–2019: a systematic analysis for the Global Burden of Disease Study 2019. Lancet Gastroenterol Hepatol.

[30] Freeland C., Racho R., Kamischke M. (2021). Health-related quality of life for adults living with hepatitis B in the United States: a qualitative assessment. J Patient Rep Outcomes.

[31] Tu T., Block J.M., Wang S. (2020). The lived experience of chronic hepatitis B: a broader view of its impacts and why we need a cure. Viruses.

[32] Freeland C., Farrell S., Kumar P. (2021). Common concerns, barriers to care, and the lived experience of individuals with hepatitis B: a qualitative study. BMC Public Health.

[33] Wong R.J., Kaufman H.W., Niles J.K. (2023). Simplifying treatment criteria in chronic hepatitis B: reducing barriers to elimination. Clin Infect Dis.

[34] Alshuwaykh O., Daugherty T., Cheung A. (2022). Incidence of hepatocellular carcinoma in chronic hepatitis B virus infection in those not meeting criteria for antiviral therapy. Hepatol Commun.

[35] Tan S.H.S., Wang D., Tan W.J. (2020). Facilitators and barriers of Hepatitis B screening and vaccination. Vaccine.

[36] Freeland C., Bodor S., Perera U. (2020). Barriers to hepatitis B screening and prevention for African immigrant populations in the United States: a qualitative study. Viruses.

[37] Juday T., Tang H., Harris M. (2011). Adherence to chronic hepatitis B treatment guideline recommendations for laboratory monitoring of patients who are not receiving antiviral treatment. J Gen Intern Med.

[38] Yao K., Liu J., Wang J. (2021). Distribution and clinical characteristics of patients with chronic hepatitis B virus infection in the grey zone. J Viral Hepat.

[39] Huang R., Do A.T., Toyoda H. (2025 Aug). Distribution, characteristics, and natural history of diverse types of indeterminate chronic hepatitis B: a real-B study. Aliment Pharmacol Ther.

[40] Di Bisceglie A.M., Lombardero M., Teckman J. (2017). Determination of hepatitis B phenotype using biochemical and serological markers. J Viral Hepat.

[41] Mak L.Y., Yee L.J., Wong R.J. (2024). Hepatocellular carcinoma among patients with chronic hepatitis B in the indeterminate phase. J Viral Hepat.

[42] European Association for the Study of the Liver (2017 Aug). EASL 2017 Clinical Practice Guidelines on the management of hepatitis B virus infection. J Hepatol.

[43] Tran T.T. (2011). Immune tolerant hepatitis B: a clinical dilemma. Gastroenterol Hepatol (N Y).

[44] Huang R., Liu J., Wang J. (2024). Histological features of chronic hepatitis B patients with normal alanine aminotransferase according to different criteria. Hepatol Commun.

[45] Zhou K., Terrault N. (2018). Immune tolerant HBV and HCC: time to revise our tolerance levels for therapy?. AME Med J.

[46] Mason W.S., Gill U.S., Litwin S. (2016 Nov). HBV DNA integration and clonal hepatocyte expansion in chronic hepatitis B patients considered immune tolerant. Gastroenterology.

[47] Protzer U., Knolle P. (2016). “To Be or not to Be”: immune tolerance in chronic hepatitis B. Gastroenterology.

[48] McMillan S.S., King M., Tully M.P. (2016). How to use the nominal group and Delphi techniques. Int J Clin Pharm.

[49] Niederberger M., Spranger J. (2020). Delphi technique in health sciences: a map. Front Public Health.

[50] Polisetty R.S., Borkowski J., Georges D. (2022). Nothing about me without me: shared decision-making in chronic hepatitis B. EMJ Hepatol.

[51] World Health Organization (2022). Global health sector strategies on, respectively, HIV, viral hepatitis and sexually transmitted infections for the period 2022-2030. הארץ.

[52] Toumi M., Wallace J., Cohen C. (2024). Experience and impact of stigma in people with chronic hepatitis B: a qualitative study in Asia, Europe, and the United States. BMC Public Health.

[53] Tu T., Block J.M., Wang S. (2020). The lived experience of chronic hepatitis B: a broader view of its impacts and why we need a cure. Viruses.

[54] Tu T., Yussf N., Tran L. (2025). Best practices for engaging with affected communities: chronic hepatitis B as a case study. Infect Dis Poverty.

[55] Lok J., Dusheiko G., Carey I. (2022 Sep). Review article: novel biomarkers in hepatitis B infection. Aliment Pharmacol Ther.

[56] Wallace J., McNally S., Richmond J. (2011 Mar 3). Managing chronic hepatitis B: a qualitative study exploring the perspectives of people living with chronic hepatitis B in Australia. BMC Res Notes.

[57] Richmond J., Smith E., Wallace J. (2017). Hepatitis B testing and diagnosis experiences of patients and primary care professionals in Australia. Aust Fam Physician.

[58] Ghany M.G., Buti M., Lampertico P. (2023). Guidance on treatment endpoints and study design for clinical trials aiming to achieve cure in chronic hepatitis B and D: report from the 2022 AASLD-EASL HBV-HDV Treatment Endpoints Conference. J Hepatol.

[59] Kang H., Lok A.S.F. (2006). Endpoints for clinical trials on treatment of hepatitis C. J Hepatol.

[60] Lok ASF (2024). Toward a Functional Cure for Hepatitis B. Gut Liver.

[61] Newton L., Necochea R., Palm D. (2019). Revisiting the “sterilising cure” terminology: a call for more patient-centred perspectives on HIV cure-related research. J Virus Erad.

[62] Dilmitis S., Edwards O., Hull B. (2012). Language, identity and HIV: why do we keep talking about the responsible and responsive use of language? Language matters. J Int AIDS Soc.

[63] Hepatitis B Foundation (2020). https://www.hepb.org/assets/Uploads/ExPFDD-Report-HBF-9-29-2020.pdf.

[64] Feld J.J., Gehring A.J., Zoulim F. (2025). Getting to HBV Cure – will new biomarkers help?. Hepatology.

